# Cyclin E2 Promotes Whole Genome Doubling in Breast Cancer

**DOI:** 10.3390/cancers12082268

**Published:** 2020-08-13

**Authors:** Christine Lee, Kristine J. Fernandez, Sarah Alexandrou, C. Marcelo Sergio, Niantao Deng, Samuel Rogers, Andrew Burgess, C. Elizabeth Caldon

**Affiliations:** 1 The Kinghorn Cancer Centre, Garvan Institute of Medical Research, Sydney, NSW 2010, Australia; c.lee@garvan.org.au (C.L.); k.fernandez@garvan.org.au (K.J.F.); s.alexandrou@garvan.org.au (S.A.); m.sergio@garvan.org.au (C.M.S.); n.deng@garvan.org.au (N.D.); 2 St. Vincent’s Clinical School, Faculty of Medicine, UNSW Sydney, Sydney, NSW 2052, Australia; 3 Children’s Medical Research Institute, The University of Sydney, Westmead, NSW 2145, Australia; srogers@cmri.org.au; 4 Faculty of Medicine and Health, University of Sydney, Sydney, NSW 2006, Australia; 5 ANZAC Research Institute, Concord, NSW 2139, Australia; andrew.burgess@sydney.edu.au; 6 The University of Sydney Concord Clinical School, Faculty of Medicine and Health Concord, Sydney, NSW 2139, Australia

**Keywords:** cyclin E2, cyclin E1, genome doubling, genomic instability, p53

## Abstract

Genome doubling is an underlying cause of cancer cell aneuploidy and genomic instability, but few drivers have been identified for this process. Due to their physiological roles in the genome reduplication of normal cells, we hypothesised that the oncogenes cyclins E1 and E2 may be drivers of genome doubling in cancer. We show that both cyclin E1 (*CCNE1*) and cyclin E2 (*CCNE2*) mRNA are significantly associated with high genome ploidy in breast cancers. By live cell imaging and flow cytometry, we show that cyclin E2 overexpression promotes aberrant mitosis without causing mitotic slippage, and it increases ploidy with negative feedback on the replication licensing protein, Cdt1. We demonstrate that cyclin E2 localises with core preRC (pre-replication complex) proteins (MCM2, MCM7) on the chromatin of cancer cells. Low *CCNE2* is associated with improved overall survival in breast cancers, and we demonstrate that low cyclin E2 protects from excess genome rereplication. This occurs regardless of p53 status, consistent with the association of high cyclin E2 with genome doubling in both p53 null/mutant and p53 wildtype cancers. In contrast, while cyclin E1 can localise to the preRC, its downregulation does not prevent rereplication, and overexpression promotes polyploidy via mitotic slippage. Thus, in breast cancer, cyclin E2 has a strong association with genome doubling, and likely contributes to highly proliferative and genomically unstable breast cancers.

## 1. Background

Genome doubling occurs in ~30–37% of primary cancers [1,2], and up to 56% of metastatic cancers [3]. A doubled genome provides an advantage to cancer cells by increasing tolerance to chromosomal instability [4], and hence increasing aneuploidy, cancer cell heterogeneity and intrinsic resistance to therapy [5,6]. Whole genome doubling leads to cancers with two-fold higher driver amplification events [3] and is significantly associated with poorer overall survival [1].

Cancer subtypes differ in their associated genomic drivers of whole genome doubling, but the drivers for each cancer subtype are poorly characterised [1]. A known driver is cyclin E1 [1,2], which is particularly associated with polyploidy in high-grade serous ovarian cancer [7]. Cyclin E1 belongs to a family of two E-cyclin proteins, cyclins E1 and E2. These are highly conserved cell cycle regulatory proteins that activate CDK2 to promote cell cycle progression. In addition, cyclins E1 and E2 have crucial roles in the normal development of differentiated cells that undergo whole genome doubling to become polyploid. Cyclin E1-/- E2-/- double knockout mice are embryonic lethal due to defects in endoreduplication of the giant trophoblasts of the placenta [8]. When rescued via tetraploid complementation, the megakaryocytes of cyclin E1-/- E2-/- double knockout embryos fail to accumulate high DNA content [8,9].

As well as its role in the normal cell cycle, cyclin E1 is a potent oncogene: it is overexpressed or amplified in multiple malignancies, expression correlates with decreased survival [10] and transgenic mice with deregulated cyclin E1 expression develop carcinomas [11]. Underpinning the oncogenic properties of cyclin E1 are its abilities to increase cell proliferation and induce genomic instability. Cyclin E1-mediated genomic instability occurs through replication stress, leading to the under-replication of DNA in late S phase and genome deletion [12]. Simultaneously, it inhibits the APC^Cdh1^ complex during mitosis, causing the misalignment of chromosomes at the metaphase plate, which results in chromosome missegregation and polyploidy [13].

Cyclin E2 is less studied than cyclin E1, but it is also pro-proliferative, promotes genomic instability and high expression correlates with poor patient outcome in multiple malignancies [14]. While cyclin E1 and E2 are often assumed to be interchangeable, there are key differences in oncogenesis. In breast cancer, cyclin E2 shows a greater prognostic value, including being part of gene signatures that predict disease progression in metastatic breast cancer, whereas cyclin E1 is absent from these same signatures [15,16]. Cyclin E2 has greater S phase stability [17], and is independently transcribed and degraded [18]. Finally, while both cyclins E1 and E2 can induce genomic instability, cyclin E2 does not inhibit the APC^Cdh1^ complex [19], and the mechanistic basis for how it induces genomic instability is unknown.

Here, we explore the roles of cyclins E1 and E2 in genome doubling in breast cancer. We identify that cyclin E2, unlike cyclin E1, induces inappropriate genome rereplication in breast cancer cells to drive polyploidy.

## 2. Results

### 2.1. Cyclin E2 Expression and Amplification Is Higher in Breast Cancers that Have Undergone Genome Doubling

**Figure 1:**
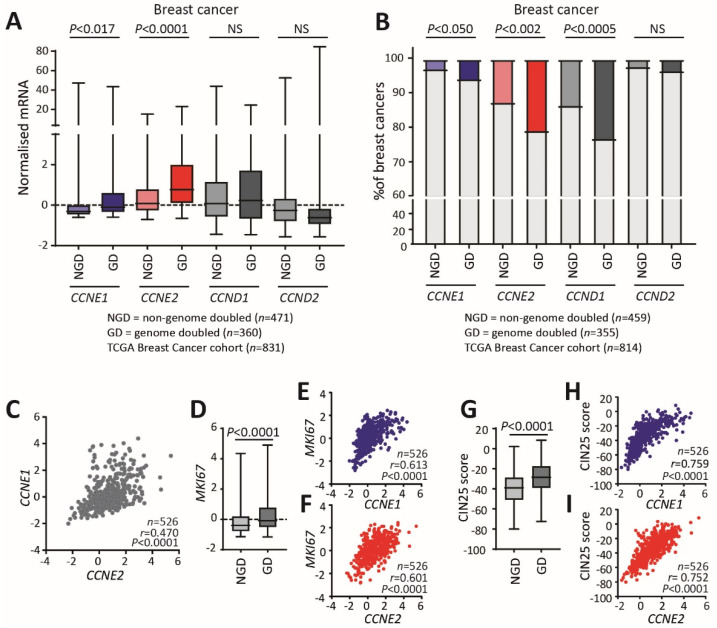
Cyclin E1 and cyclin E2 are associated with whole genome doubling in breast cancer. (**A**) Relative *CCNE1*, *CCNE2*, *CCND1*, *CCND2* expression was determined across the TCGA breast cancer dataset and compared between non-genome doubled (NGD) and genome doubled (GD) cancers. Data were analysed by Welch’s *t*-test. (**B**) Relative *CCNE1*, *CCNE2*, *CCND1*, *CCND2* amplification was determined across the TCGA breast cancer dataset and compared between non-genome doubled (NGD) and genome doubled (GD) cancers. Data were analysed by Fisher’s exact test. (**C**) Scatter plots of *CCNE1* and *CCNE2* gene expression across breast cancers. Axes show log intensity level z-scores, r is Pearson co-efficient. (**D**) *MKI67* gene expression (z-score) in NGD and GD breast cancers. Data were analysed by Welch’s *t*-test. E/F. Scatter plots of (**E**) *CCNE1* versus *MKI67* gene expression (z-score) and (**F**) *CCNE1* versus *MKI67* gene expression (z-score) across breast cancers, r is Pearson co-efficient. (**G**) CIN25 gene expression score in NGD and GD breast cancers. Data were analysed by Welch’s *t*-test. H/I. Scatter plots of (**H**) *CCNE1* versus CIN25 and (**I**) *CCNE2* versus CIN25 across breast cancers, r is Pearson co-efficient. Gene expression data were downloaded from TCGA (cBioPortal, http://cbioportal.org).

We first determined how cyclin E1 and cyclin E2 related to genome ploidy by examining the relationship between the expression of the cyclins E1 and E2 genes, *CCNE1* and *CCNE2*, and the genome doubling status of TCGA breast cancers (*n* = 831). We also examined the relationship to genome doubling of the *CCND1* and *CCND2* genes for cyclins D1 and D2, as these have functional overlap with cyclins E1 and E2 in driving G_1_ to S phase cell cycle progression. Genome doubling was determined by ASCAT (Allele Specific Copy Number Analysis of Tumours) analysis to identify the number of whole genomes, and the demarcation between non-doubled and doubled genomes can be clearly identified at 2.7 genome copies (Figure S1A). Of the four cyclins, *CCNE1* and *CCNE2* mRNA occurred at significantly higher levels in whole genome doubled (GD) tumours versus non-genome doubled (NGD) tumours in breast cancers (Figure 1A).

Cyclin E1 drives polyploidy in high-grade serous ovarian cancer in association with *CCNE1* gene amplification [7] and is generally associated with genome doubling [1], but cyclin E2 has not been reported in this context. When we analysed the ploidy of TCGA ovarian cancers, we found, as expected, that *CCNE1* occurred at significantly higher levels in whole genome doubled tumours versus non-genome doubled, although *CCNE2* did not (Figure S2A).

We then examined the relationship between gene amplification and genome doubling in breast cancer, by comparing *CCNE1*, *CCNE2*, *CCND1* and *CCND2* gene amplification in non-genome doubled and genome doubled tumours. *CCNE1* gene amplification is rare in breast cancer (3.4%), whereas *CCNE2* gene amplification occurs in 16.4% of breast cancers. A small number of cancers show amplification of both genes (0.6%). *CCNE1* and *CCND1* gene amplification was significantly higher in genome doubled cancers, as has been previously observed (Figure 1B). However, we made the novel observation that *CCNE2* amplification was also significantly enhanced in genome doubled breast cancers (Figure 1B). 

*CCNE1* and *CCNE2* are generally assumed to have highly overlapping regulation and functions [14], but we examined the relationship between *CCNE1* and *CCNE2* mRNA expression and found that they are not highly correlated in breast cancers (Figure 1C). Both *CCNE1* and *CCNE2* are reported as related to proliferation and genomic instability, which are two features of genome doubled cancers [1]. This led us to question if *CCNE1* and *CCNE2* have similar relationships with proliferation and genome instability in breast cancer. *MKI67*, the gene which encodes the proliferation-associated protein Ki67 has significantly higher expression in genome doubled cancers (Figure 1D). We found that *CCNE1* and *CCNE2* had similar strong correlations to *MKI67* (*CCNE1* vs. *MIK67*: r = 0.613; *CCNE2* vs. *MKI67*: r = 0.601; Figure 1E,F), indicating that, although they are not strongly correlated to each other, they both correlate with proliferation.

We then demonstrated that genome doubled cancers have a significantly higher CIN25 chromosomal instability index, a gene expression signature that includes the 25 genes most highly correlated with aneuploidy [20] (Figure 1G). We found that *CCNE1* and *CCNE2* had similar strong correlations to the CIN25 index (*CCNE1* vs. CIN25: r = 0.759; *CCNE2* vs. CIN25: r = 0.752; Figure 1H,I).

### 2.2. Survival Analysis in Genome Doubled and Non Genome Doubled Cancers Based on CCNE1 and CCNE2 Expression and Amplification

**Figure 2:**
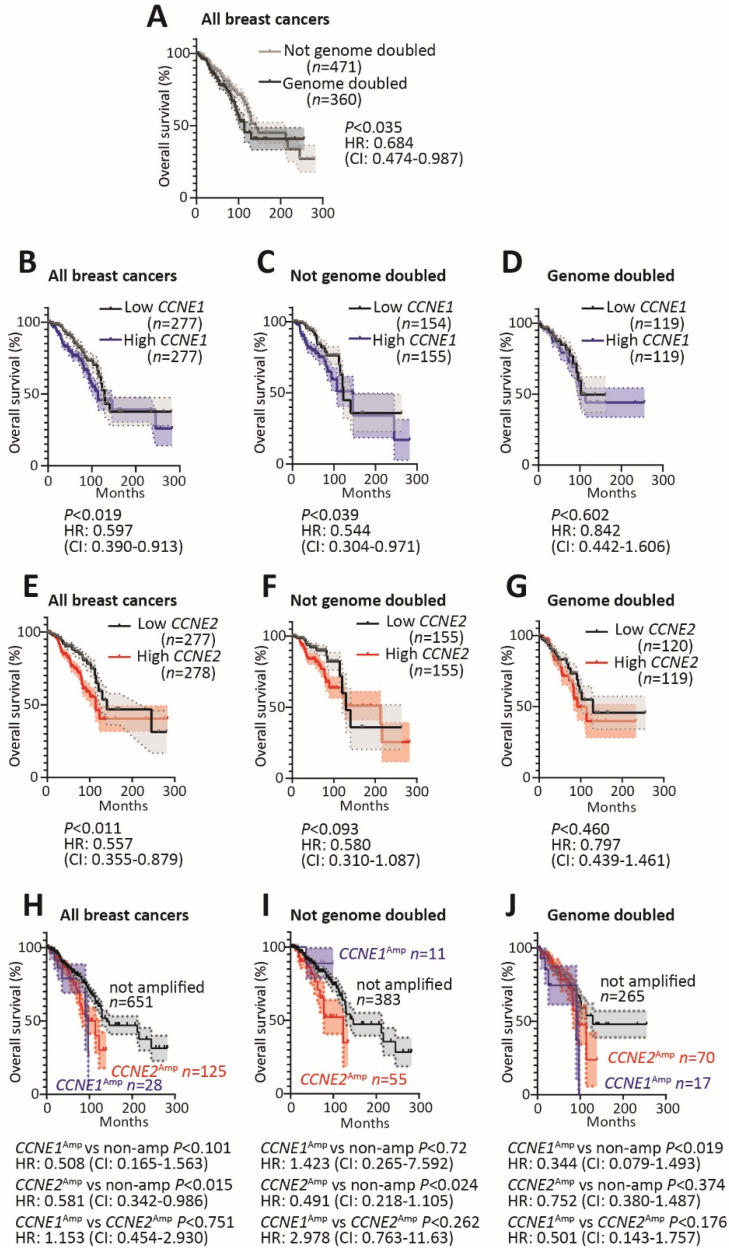
Survival analysis in genome doubled and non-genome doubled cancers based on *CCNE1* and *CCNE2* expression and amplification. For all analyses, gene expression and amplification data and survival outcomes for breast cancer were downloaded from TCGA (cBioPortal, http://cbioportal.org). The *p*-values are calculated by logrank Kaplan–Meier (K–M) analyses and hazard ratios from the logrank test. (**A**) K–M curves show estimated survival over time associated with genome doubling in breast cancers. NGD is non-genome doubled (*n* = 471) and GD is genome doubled (*n* = 360). (**B**) K–M curves of estimated survival in all breast cancers associated with high *CCNE1* (top tertile) and low *CCNE1* (bottom tertile). (**C**) K–M curves of estimated survival in NGD breast cancers associated with high *CCNE1* (top tertile) and low *CCNE1* (bottom tertile). (**D**) K–M curves of estimated survival in GD breast cancers associated with high *CCNE1* (top tertile) and low *CCNE1* (bottom tertile). (**E**) K–M curves of estimated survival in all breast cancers associated with high *CCNE2* (top tertile) and low *CCNE2* (bottom tertile). (**F**) K–M curves of estimated survival in NGD breast cancers associated with high *CCNE2* (top tertile) and low *CCNE2* (bottom tertile). (**G**) K–M curves of estimated survival in GD breast cancers associated with high *CCNE2* (top tertile) and low *CCNE2* (bottom tertile). (**H**) K–M curves of estimated survival associated with *CCNE1* amplification (*n* = 28), *CCNE2* amplification (*n* = 125) and cancers without amplification of *CCNE1*/*CCNE2* (*n* = 651). (**I**) K–M curves of estimated survival in NGD breast cancers associated with *CCNE1* amplification (*n* = 11), *CCNE2* amplification (*n* = 55) and cancers without amplification of *CCNE1*/*CCNE2* (*n* = 383). (**J**) K–M curves of estimated survival in GD breast cancers associated with *CCNE1* amplification (*n* = 17), *CCNE2* amplification (*n* = 70) and cancers without amplification of *CCNE1*/*CCNE2* (*n* = 265).

Genome doubling is an important facilitator of cancer evolution and it is associated with poorer prognosis and resistance to chemotherapy [1]. We confirmed that genome doubled cancers have significantly worse overall survival across the TCGA breast cancer dataset (Figure 2A). We then examined whether *CCNE1* and *CCNE2* expression has prognostic value depending on whether a cancer had undergone whole genome doubling. We found that breast cancers high in *CCNE1* had significantly worse prognosis in breast cancers (Figure 2B), and that poor prognosis was also predicted by high *CCNE1* expression in the subset of cancers that were not genome doubled (Figure 2C). High cyclin E1 did not associate with poor prognosis in already genome doubled cancers (Figure 2D). Similarly, breast cancers high in *CCNE2* had worse overall survival (Figure 2E), and a trend towards worse overall survival if they had not undergone genome doubling (Figure 2F), but not in those breast cancers which were genome doubled (Figure 2G). One explanation of these data could be that high expression of either cyclin E1 or cyclin E2 could precipitate genome doubling, leading to genome doubled cancers with high *CCNE1* or *CCNE2* expression, as shown in Figure 1. These observations would be best confirmed in other cohorts, but at this stage, no other ploidy annotated cohorts are available.

We also examined the effect of *CCNE1* and *CCNE2* gene amplification on survival. *CCNE1* amplification is not associated with poor overall survival, but *CCNE2* amplification is significantly associated with poor survival (Figure 2H). When the cohort is split in to non-genome doubled and genome doubled cancers, *CCNE2* amplification is associated with poor prognosis in those cancers that have not undergone genome doubling (Figure 2I). Of interest, *CCNE1* amplification was not associated with poor survival in non-genome doubled cancers, but instead in those that had already undergone genome doubling (Figure 2J). These observations are limited by the small number of *CCNE1* amplified breast cancer cases, leading to high confidence intervals in the analyses.

### 2.3. Excess Cyclin E2 Increases Mitotic Aberrations without Increasing the Length of Mitosis

**Figure 3:**
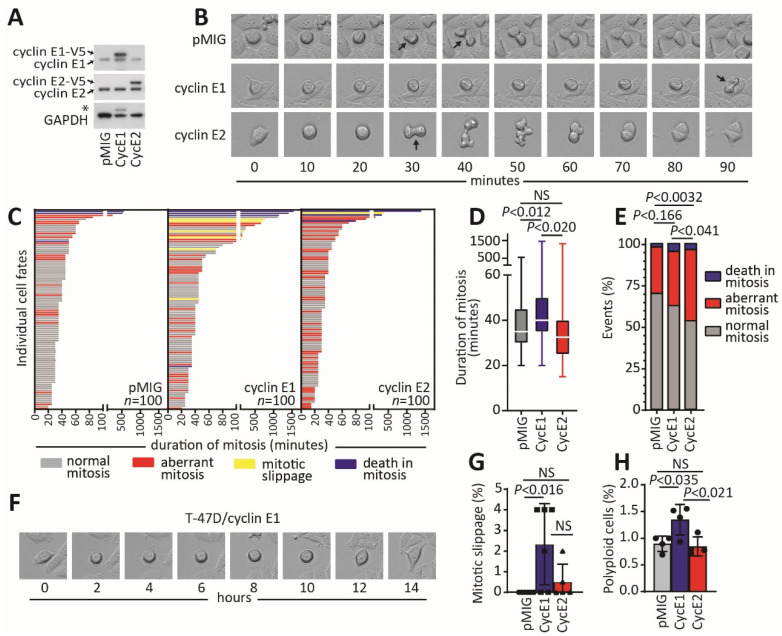
Overexpression of cyclin E2, but not cyclin E1, promotes aberrant mitosis without mitotic slippage. (**A**) T-47D cells constitutively overexpressing cyclin E1, cyclin E2 or pMIG vector were western blotted to confirm overexpression. The uncropped western blot figure in Figure S4. * Indicates cyclin E1 band in the GAPDH blot reprobed from the same membrane. (**B**) Live cell imaging was used to monitor progression of cells for 72 h through the cell cycle, representative examples of T-47D pMIG, T-47D cyclin E1 and T-47D cyclin E2 shown. Arrows indicate anaphase. (**C**) Quantitation of mitoses of 100 cells per cell line. Grey are normal mitoses, red are aberrant mitoses (multipolar, asymmetrical divisions, multiple attempts at mitosis), yellow are mitotic slippages, blue are death in mitosis. D/E. Live cell imaging was scored over four fields of view in duplicate experiments for each cell line. (**D**) shows that the duration from nuclear envelope breakdown to anaphase. Data were analysed by one-way ANOVA, with Tukey’s multiple comparisons test. (**E**) The number of normal mitoses, aberrant mitoses and deaths in mitosis. Data were analysed by chi-squared test; NS is not significant. (**F**) Example of T-47D-cyclin E1 cell undergoing mitotic slippage. (**G**) Quantitation of mitotic slippage in each cell line scored over four fields of view in duplicate experiments. Data were analysed by one-way ANOVA, with Tukey’s multiple comparisons test; NS is not significant. (**H**) Quantitation of polyploid cells by flow cytometry of propidium iodide-stained cells gated for >4N content. Data were analysed by one-way ANOVA, with Tukey’s multiple comparisons test; NS is not significant.

Since our data suggest that both cyclins E1 and E2 are associated with aneuploid development, we compared the effect of high cyclins E1 and E2 on aberrant cell divisions that could result in a doubled genome. A doubled genome occurs in association with deregulated transit through mitosis to the following cell cycle, occurring via mitotic slippage or rereplication of the genome. We examined T-47D breast cancer cells stably overexpressing cyclin E1-V5, cyclin E2-V5 or a vector control (pMIG - pMSCV-IRES-GFP) (Figure 3A). Cells were previously sorted based on co-expression of the GFP protein and V5 expression into subpopulations of cells that had similar moderate overexpression of protein in each cell line [19].

We first determined the time to transit through mitosis (Figure 3B,C). Vector control and cyclin E2 overexpressing cells had similar transit times (pMIG: 50.95 min and cyclin E2: 55.6 min; *p* < 0.983) (Figure 3D). By contrast, T-47D cells overexpressing cyclin E1 had significantly longer transit (cyclin E1:126.0 min; *p* < 0.01) (Figure 3D). Cells overexpressing cyclin E1 also had a trend towards an increase in aberrant mitotic events (Figure 3E). In particular, cyclin E1 overexpressing cells had an increased frequency of mitotic slippage, where cells would enter mitosis, but then exit without undergoing a cell division (Figure 3F,G). We also examined the abundance of polyploid cells in the cell lines, and we found that cyclin E1 overexpressing cells had a higher basal level of polyploid cells than either control cells or cyclin E2 overexpressing cells (Figure 3H).

While cells overexpressing cyclin E2 did not have increased mitotic length, they did have a significant increase in the number of aberrant mitoses compared to both control cells and cyclin E1 overexpressing cells (Figure 3E), such as the multipolar mitosis shown in Figure 3B. It was notable that cyclin E2 overexpressing cells did not show significantly increased mitotic slippage or polyploidy compared to the vector control (Figure 3G,H), although they did have increased death in mitosis (cyclin E2: 4%; vector control: 2%).

**Figure 4:**
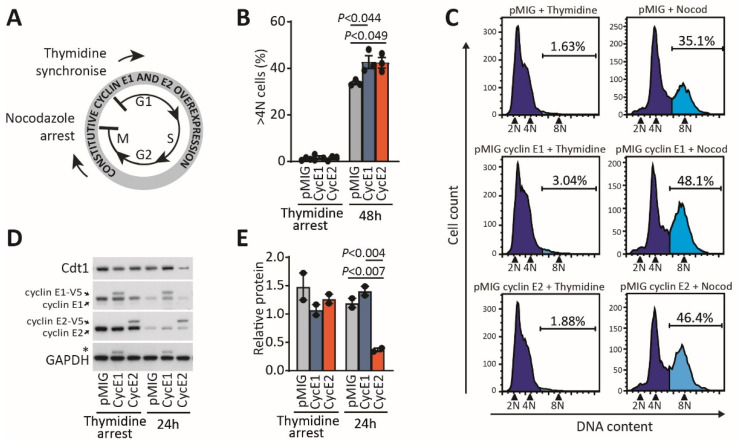
Overexpression of cyclin E2 promotes polyploidy in association with Cdt1 downregulation. (**A**) Schematic of nocodazole-mediated arrest in T-47D cells constitutively expressing cyclins E1 and E2: cells are arrested at G_1_ with thymidine, released into the cell cycle and then arrested at mitosis with nocodazole. (**B**) Quantitation of polyploid cells after nocodazole arrest by flow cytometry of propidium iodide-stained cells gated for >4N content. N = sets of chromosomes. Data were analysed by one-way ANOVA, with Tukey’s multiple comparisons test. (**C**) Representative examples are shown of each cell line after nocodozole arrest, where they are stained with propidium iodide and analysed by flow cytometry to detect >4N cells. N = sets of chromosomes. (**D**) Western blotting for cyclin E1, cyclin E2, Cdt1 and GAPDH of cell lysates from cells synchronised with thymidine, and then arrested for 24 h with nocodazole. The uncropped western blot figure in Figure S4. (**E**) Quantitation of Cdt1 expression by densitometry of duplicate experiments, error bars indicate range. Data were analysed by one-way ANOVA, with Tukey’s multiple comparisons test.

Since we had not observed any increase in polyploidy from cyclin E2 overexpression associated with mitotic slippage, we then determined whether cyclins E1 and E2 gene expression could induce polyploidy through other mechanisms. Rare polyploid events are more easily detected in synchronised cells following mitotic arrest [21]. We first synchronised cells at G_1_ with thymidine, followed by prolonged block at mitosis with nocodazole (Figure 4A). T-47D cells express mutant p53, hence eliminating the tetraploid checkpoint [22] and improving the survival of tetraploid cells following their induction. Using flow cytometry, we observed that, compared to T-47D control cells, both cyclins E1 and E2 overexpressing cells had higher levels of 4N–8N DNA content 48 h after nocodazole arrest compared to the pMIG control (Figure 4B,C). Cyclin E2 overexpressing cells did not show increased polyploidy compared to the basal levels of polyploidy that we observed with cyclin E1 overexpression (Figure 3H).

In the absence of induced mitotic slippage events (Figure 3), we hypothesised that cyclin E2 induces a new round of genome replication following mitotic arrest. We thus performed western blots for Cdt1 in the nocodazole-treated cells. Cdt1 is the replication initiation factor that licences a new round of DNA replication via the pre-replication complex (preRC), and it is degraded in response to inappropriate DNA rereplication [23]. We found that, with cyclin E2 overexpression, Cdt1 was significantly depleted at 24 h following nocodazole arrest of the cells, but Cdt1 levels remained unchanged with cyclin E1 overexpression (Figure 4D,E). This suggests that cyclin E2 induces DNA rereplication when overexpressed in T-47D breast cancer cells.

### 2.4. Cyclin E2 Preferentially Localises to Chromatin during Endoreduplication

In quiescent fibroblasts, both cyclins E1 and E2 interact with the preRC [24] but, paradoxically, cyclin E1 overexpression interferes with preRC assembly in cancer cells [25]. Consequently, we compared how cyclins E1 and E2 interact with the preRC in cancer cells that are undergoing genome doubling. For these experiments, we used the well-established and tightly synchronous model of the genome duplication of HeLa cells induced by treatment with the CDK1 inhibitor RO3306 [26]. CDK1 suppresses origin licencing in G_2_ via dual inhibitory actions on the preRC: it phosphorylates the origin replication complex and sterically inhibits the complex to prevent the recruitment of minichromosome maintenance (MCM) proteins [27]. Since CDK1 inhibition blocks cells in G_2_ phase, this model is termed endoreduplication, as the cells re-enter the cell cycle before reaching mitosis.

**Figure 5:**
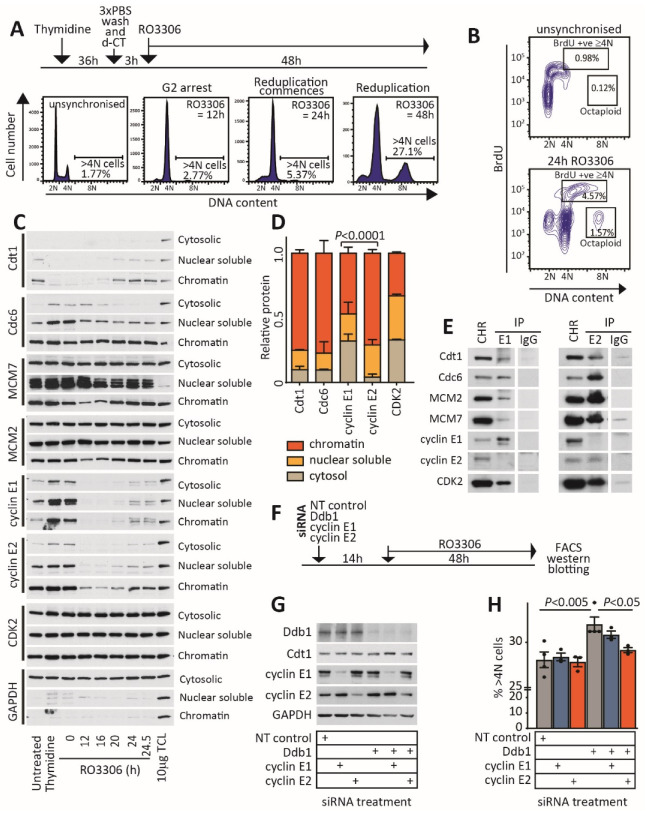
Cyclin E2/CDK2 localises to chromatin of endoreduplicating cells, and cyclin E2 depletion abrogates endoreduplication. (**A**) HeLa cells synchronised with thymidine were treated for 3 h with deoxycytidine (d-CT), subsequently treated with RO3306 and were then monitored by flow cytometry for the commencement of endoreduplication. (**B**) DNA replication was confirmed by BrdU incorporation, where cells were pulsed with BrdU at 24 h post RO3306 addition for 15 min. N = sets of chromosomes. (**C**) Cytosolic, nuclear soluble and chromatin lysates were collected at intervals following RO3306 addition, and western blotted for the preRC proteins Cdt1, Cdc6, MCM2, MCM7 and for cyclin E1, cyclin E2, CDK2 and GAPDH. TCL = total cell lysate. The uncropped western blot figure in Figures S5–S7. (**D**) The relative proportion of protein in each cell fraction was quantitated by ImageJ, and compared with a chi-squared test. (**E**) Purified chromatin lysates were immunoprecipitated with IgG, cyclin E1 or cyclin E2 and immunoprecipitates were western blotted for preRC proteins and CDK2. The uncropped western blot figure in Figures S8 and S9. (**F**) HeLa cells were transfected with siRNAs to Ddb1 and a non-targeting control or siRNAs to cyclin E1 or cyclin E2. After 14 h, cells were treated with 10 µM RO3306, and cells were collected 48 h later for analysis for >4N cells by flow cytometry and by western blotting. (**G**) Western blotting of siRNA-treated cells for Ddb1, Cdt1, cyclin E1, cyclin E2 and GAPDH. (**H**) Quantitation of >4N cells following siRNA/RO3306 treatment. Treatments were compared by one-way ANOVA, followed by Tukey’s multiple comparisons. The uncropped western blot figure in Figure S10.

We examined the localisation of cyclins E1 and E2 to chromatin in RO3306-treated HeLa cells. Cells were collected at intervals up to 24 h following mid-S phase (0 h). The commencement of endoreduplication at 20–24 h was confirmed by the identification of an 8N population by flow cytometry (Figure 5A), BrdU incorporation within the polyploid population (Figure 5B) and the accumulation of the preRC protein Cdt1 on chromatin (Figure 5C). To determine the location of cyclin E1 and E2 during endoreduplication, cell lysates were separated into cytosol, nuclear soluble and chromatin fractions (Figure 5C). These showed that cyclins E1 and E2 expression increased at 24 h post RO3306 when cells were poised for endoreduplication, but while cyclin E1 was distributed throughout the cell, 71% of cyclin E2 was predominantly loaded on chromatin, much like the preRC components Cdc6 and Cdt1 (75% and 77%, respectively) (Figure 5C,D). Altogether, this indicates that cyclin E2 specifically accumulates on the chromatin of cells poised for endoreduplication.

We subsequently examined the association of cyclins E1 and E2 with preRC components. Chromatin was extracted following 24 h of RO3306 to induce endoreduplication, and cyclins E1 and E2 were immunoprecipitated from the chromatin. Both cyclins E1 and E2 bound multiple preRC components, including Cdt1, Cdc6, MCM2 and MCM7. However, compared to cyclin E1, cyclin E2 had a particularly enhanced association with MCM2 and MCM7 in the chromatin of cells arrested with RO3306 (Figure 5E).

### 2.5. Depletion of Cyclin E2, But Not Cyclin E1, Ablates Rereplication

While we saw a difference in the association between cyclins E1 and E2 with MCM2 and MCM7 by immunoprecipitation, this may be due to differences in the affinity of complexes to the cyclins E1 and E2 antibodies, rather than differences in the complexes themselves. For this reason, we compared the effect of the loss of cyclin E1 and cyclin E2 protein on the induction of endoreduplication. Due to toxicity, we were not able to simultaneously synchronise cells with thymidine and treat with siRNAs, so we instead transfected cells with siRNAs to cyclin E1, cyclin E2 and a non-targeting control, followed by a 14-h incubation with the CDK1 inhibitor RO3306 (Figure 5F). Treatment with cyclin E1 and E2 siRNAs did not lead to any changes in the proportion of >4N cells compared to a non-targeting (NT) control (Figure 5G,H).

We then further exacerbated endoreduplication by treatment with Ddb1 siRNA. Ddb1 is part of the Ddb1-Cul4 ubiquitin ligase complex that targets Cdt1 [28], and the loss of Ddb1 reduces Cdt1 turnover and induces excess DNA rereplication. Ddb1 siRNA treatment enhanced RO3306-induced polyploidy (Figure 5H). When cyclin E2 siRNA was combined with Ddb1 siRNA, the increase in polyploidy was abrogated (Figure 5H). By contrast, combination cyclin E1/Ddb1 siRNA treatment did not abrogate polyploidy (Figure 5H). Thus, the depletion of cyclin E2 can attenuate excess endoreduplication when the regulation of Cdt1 is compromised.

### 2.6. Cyclin E2 Is Associated with Genome Doubling in p53 Wildtype Breast Cancers, and Its Depletion in p53 Wildtype Breast Cancer Cells Reduces DNA Rereplication

T-47D and HeLa cells both lack normal p53 function, as T-47D cells express mutant p53 and HeLa cells lack p53 due to expression of the viral E6 protein. The loss of normal p53 function prevents cells from detecting and eliminating genome doubled cells, known as the “tetraploidy checkpoint” [29]. Genome doubling is thus more prevalent in p53 disrupted cancers, but 46% of genome doubling events occur in p53 wildtype cancers [1]. This led us to question whether *CCNE2* was associated with genome doubling in both p53 null/mutant and p53 wildtype cancers, as ~60% of breast cancers have wildtype p53 [30].

**Figure 6:**
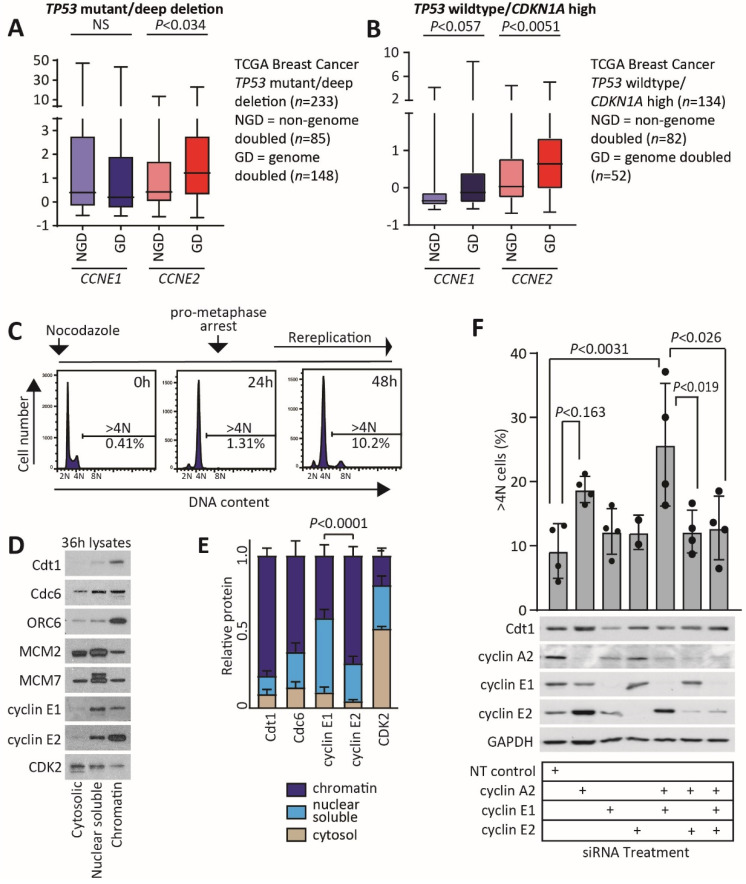
Cyclin E2 is associated with genome doubling in p53 wildtype breast cancers. (**A**) Relative *CCNE1* and *CCNE2* expression was determined across the TCGA breast cancer dataset and compared between non-genome doubled (NGD) and genome doubled (GD) cancers with *TP53* mutation/deep deletion. Data were analysed by Welch’s *t*-tests. (**B**) Relative *CCNE1* and *CCNE2* expression was determined across the TCGA breast cancer dataset and compared between non-genome doubled (NGD) and genome doubled (GD) cancers with wildtype *TP53* status, high *TP53* expression and high *CDKN1A* expression. Data were analysed by Welch’s *t*-tests. (**C**) Schematic of nocodazole-mediated arrest in MCF-7 cells and effect on cell cycle distribution at 0 h, 24 h and 48 h, determined by propidium iodide staining of cells and detection of >4N cells by flow cytometry. N = sets of chromosomes. (**D**) Cytosolic, nuclear soluble and chromatin lysates were collected at 36 h following nocodazole addition, and western blotted for the preRC proteins Cdt1, Cdc6, ORC6, MCM2, MCM7 and for cyclin E1, cyclin E2 and CDK2. (**E**) The relative proportion of protein in each cell fraction was quantitated by ImageJ, and compared with a chi-squared test. (**F**) MCF-7 cells transfected with siRNAs to cyclin A2, cyclin E1, cyclin E2 and non-targeting control were blocked at pro-metaphase with 50 ng/mL nocodazole, and cells were collected 48 h later for analysis for >4N cells by flow cytometry and by western blotting. Treatments were compared by one-way ANOVA, with Tukey’s multiple comparisons test. The uncropped western blot figure in Figure S11.

First, we examined the relationship between *CCNE1*, *CCNE2* and genome doubling in p53 null/mutant breast cancers. We defined p53 null/mutant cancers from the TCGA breast cancer dataset as those with a deep deletion of *TP53* concomitant with very low *TP53* mRNA expression, or having a deleterious mutation of *TP53*. *CCNE2* was significantly higher in p53 null/mutant breast cancers with genome doubling than in those with a near-diploid genome. *CCNE1* was not higher in p53 null/mutant breast cancers that were genome doubled (Figure 6A).

The p53 functional breast cancers were defined based on *TP53* status and the expression of p21. First we identified p53 wildtype patients in the TCGA breast cancer dataset that lacked *TP53* mutation, lacked *TP53* deep deletion and had a *TP53* mRNA z-score > 0. As p53 function is disrupted by other mechanisms, such as a high expression of the p53-targeting proteins MDM2 and MDM4, we then defined p53 functional cancers as those which are p53 wildtype and have high p21 expression. We examined p21 gene expression (*CDKN1A*) in the p53 wildtype and p53 mutated/deep deleted cancers by downloading expression data from TCGA. There were 69 cases of p53 wildtype breast cancer that had a similar expression of p21 to p21–low p53 mutated/deep deleted cancers, suggesting that these cancers could have compromised p53 function (Figure S3). We excluded these cases to define a p53 wildtype/p21 high cancer subset. We subsequently analysed the expression of *CCNE1* and *CCNE2* expression in non-genome doubled or genome doubled cancers of p53 wildtype/p21 high cancers. *CCNE1* was not significantly different between non-genome doubled and genome doubled cancers, but there was a trend (*p* < 0.057) for higher expression in genome doubled cancers. *CCNE2* was significantly higher in genome doubled cancers (*p* < 0.0051) (Figure 6B).

Since *CCNE1* and *CCNE2* mRNAs are both expressed at high levels in genome doubled p53 wildtype/p21 high breast cancers, we examined the role of cyclin E1 and cyclin E2 in preRC formation in a p53 wildtype breast cancer model, MCF-7 cells. MCF-7 cells were blocked and synchronised at pro-metaphase by treatment for 24 h with nocodazole (Figure 6C). This is prior to replication licencing, which commences during telophase [31]. Prolonged nocodazole exposure then leads to the appearance of an 8N peak by 48 h (Figure 6C). To examine events involved in initiating rereplication, we collected lysates 36 h post nocodazole arrest, and separated into cytosolic, nuclear soluble and chromatin fractions. Specifically, we examined whether cyclins E1 and E2 co-localised with components of the preRC, including Cdc6, MCM proteins and Cdt1, the replication licencing factor for the activation of preRC complexes. We identified that cyclin E1 was predominantly in the nuclear soluble fraction (49%), whereas cyclin E2 was 71% chromatin loaded, similar to the preRC proteins Cdt1 and Cdc6 (Cdt1—79%, Cdc6—63%; Figure 6D,E).

We then examined the effect of cyclins E1 and E2 on polyploid formation by performing nocodazole arrest of MCF-7 cells in combination with cyclin depletion by siRNA treatment. Cyclins E1 and E2 siRNA did not lead to any change in the percent of polyploid cells (Figure 6F). However, we had also included cyclin A2 siRNA in this experiment, with which we observed a trend towards an increase in polyploid cells. A review of the literature showed that cyclin A2 loss increases the stability of preRC proteins: Cdt1, Cdc6, ORC1 and MCM proteins are all targeted for degradation after cyclin A2/CDK1 phosphorylation, and the loss of cyclin A thus increases preRC availability and origin firing [32].

We then used cyclins E1 and E2 siRNA in combination with cyclin A2 depletion to determine how cyclin E1/E2 loss changes polyploidy when the preRC is hyperactivated. The combination of cyclin E1 and A2 depletion led to greatly increased polyploidy (2.8-fold increase; Figure 6F). In contrast, the combination of cyclin E2 and A2 siRNA resulted in polyploidy levels similar to control treatment levels, and the combination of cyclin E1, E2 and A2 siRNAs also showed the same level of polyploidy as control treatments (Figure 6F).

Together, these data imply that the loss of cyclin E2 restrains preRC activity, although the presence of cyclin E2 is not essential for polyploidy to occur. However, we also noted that cyclin E2 levels are highest in those treatments exhibiting the most polyploidy (Figure 6F). 

## 3. Discussion

Deregulated origin licencing leads to rereplication and polyploidy, which is an important precursor of chromosomal instability in cancer progression [33]. The preRC complex, which consists of Cdt1, Cdc6, ORC proteins and the mini-chromosome maintenance complex (MCM2-7), licences origins of replication. Despite the frequency of whole genome doublings and aneuploidy in cancer, this group of proteins is rarely mutated, implying that other drivers are important [34]. We here identify that cyclin E2 is one such driver, as it associates with the preRCs of cancer cells, its upregulation increases genome doubling and its depletion attenuates the induction of DNA rereplication.

**Figure 7:**
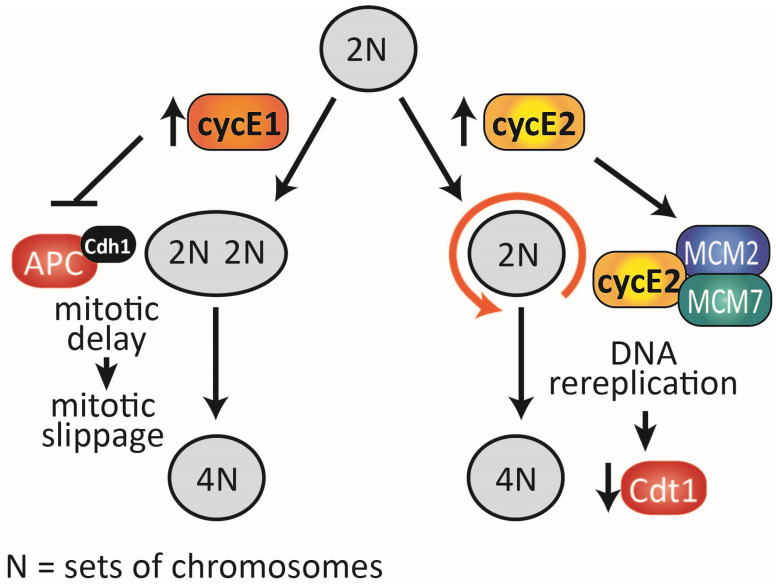
Cyclins E1 and E2 are associated with genome doubling, but through different mechanisms. Excess cyclin E1 blocks the action of the APC (Cdh1) complex [13,19], leading to delayed mitosis through a delay in the degradation of cyclin B1. A common consequence is mitotic slippage, resulting in a 4N state. Excess cyclin E2 is not associated with mitotic slippage. Instead, cyclin E2 has enhanced binding to the MCM2 and MCM7 proteins of the pre-replication complex that initiates DNA replication, and excess cyclin E2 leads to DNA rereplication. Rereplicated cells enter a 4N state, and cells downregulate Cdt1 as part of a negative feedback loop to prevent further rereplication. N = sets of chromosomes.

We previously reported that cyclin E2 overexpression induces genomic instability in association with decreased chromosome condensation and failed nuclear envelope breakdown [19], both of which feature in endoreduplicating cells [35]. Cyclin E1 is also associated with genomic instability, but this is instead likely due to established roles in inhibiting preRC complex formation [25], causing focal genomic losses through replication stress [12] and by stabilising the APC^Cdh1^ complex [13,19]. The prolonged mitosis and increased mitotic defects we observed with cyclin E1 overexpression (Figure 3) are symptomatic of these defects (Figure 7).

Our analysis of TCGA public datasets revealed that *CCNE2* amplification and mRNA are associated with poor prognosis in non-genome doubled breast cancers (Figure 1). Both *CCNE2* amplification and mRNA expression are enhanced in genome doubled breast cancers but are not associated with poor survival (Figure 2). This suggests a model where high cyclin E2 in non-genome doubled cancers increases the risk of genome doubling, leading to genome doubled cancers with high cyclin E2 expression. Our analysis of cell line models shows that the siRNA-mediated downregulation of cyclin E2 prevents the induction of polyploidy. Conversely, we also observed that the overexpression of cyclin E2 increases polyploidy, and this occurs in concert with the downregulation of the preRC protein, Cdt1 (Figure 7). Negative feedback through the proteolysis of Cdt1 is well established as a consequence of increased rereplication [36].

Recent work has identified that an E2F-mediated signature of defect in G_1_ arrest is enhanced in genome doubling independently of those cancers that have genome doubling associated with elevated *CCNE1* [1]. Cyclin E2 is a canonical E2F target gene, especially in breast cancer [18], and its overexpression is able to overcome G_1_ arrest in this setting [37]. Thus, cyclin E2 may be a component of E2F-driven genome doubling in breast cancer. This is also consistent with the frequent presence of *CCNE2* in gene signatures of breast cancer metastasis [15,16,38,39], as metastatic cancers have a higher frequency of genome doubling [3]. Cyclins E2 and D1 were also validated hits in a screen for drivers of whole chromosome instability, which is strongly associated with whole genome doubling events [40]. Surprisingly, cyclin D1 expression is not associated with genome doubling in our analysis (Figure 1A). However, this mirrors previous observations that cyclin D1 levels do not necessarily correlate with genome doubling despite promoting its tolerance [41].

A remaining question is why the two very similar genes, cyclins E1 and E2, have different mechanisms in driving genomic instability. A key difference is that cyclin E1 can be cleaved into a lower molecular weight protein that is able to induce centrosome reduplication, hence provoking genomic instability [42]. We additionally observe here, as we have reported previously [43], that cyclins E1 and E2 have very different cellular localisation patterns, where cyclin E2 is predominantly located on chromatin, and cyclin E1 is more abundant in soluble fractions of the cell. Several oncogenic functions of cyclin E1 occur in the cytosol and nuclear soluble fractions, including centrosome duplication [42]. Critically, when it was found that high cyclin E1 expression prevented the loading of MCM complexes into preRCs on chromatin to induce genomic instability, this was associated with cyclin E1 that was overexpressed in the cytosol and nuclear soluble fraction, and not loaded onto chromatin [25]. We found, in contrast, that MCM proteins bind abundantly to cyclin E2 on chromatin, providing a possible mechanism to assist preRC formation in cancer.

## 4. Methods

### 4.1. Cell Culture

MCF-7 and T-47D cells were cultured as described in [44], HeLa cells were cultured in DMEM F-12 supplemented with 10% fetal calf serum. Cell lines were cultured for <6 months after STR (Short Tandem Repeat) profiling authentication (Garvan Molecular Genetics, Garvan Institute, Sydney, NSW, Australia). Stable overexpression of cyclins E1 and E2 was achieved using infection of the pMIG vector followed by cell sorting on green fluorescent protein (GFP) expression. Derivation of T-47D pMIG, T-47D pMIG cycE1-V5 and T-47D pMIG cycE2-V5 cells has been previously described [19].

### 4.2. Synchronisation/Drug Treatments

HeLa cells synchronised at G_1_/S phase with thymidine for 36 h were released into medium supplemented with 24 μM deoxycytidine. Cells treated with 10 µM RO3306 3 h after release (t = 0 h), were collected at intervals after RO3306 treatment. MCF-7 cells were treated with 50 ng/mL nocodazole for 24 h to cause pro-metaphase arrest. T-47D cells were synchronised at G_1_/S phase with 36 h thymidine, released into medium supplemented with 24 μM deoxycytidine, and blocked at pro-metaphase with 50 ng/mL nocodazole.

### 4.3. Immunoblotting and Immunoprecipitation

Total protein, chromatin, cytosolic and nuclear soluble cell lysates were purified as described [45,46], and separated using polyacrylamide gels (Invitrogen) prior to transfer to PVDF membranes. Primary antibodies were Cdt1 (F-6), cyclin E1 (HE12), Cdc6 (180.2), CDK2 (M2), cyclin A2 (BF683) and GAPDH (6C5) from Santa Cruz Biotechnology, cyclin E2 (EP454Y) from AbCam and MCM2 (#3619), MCM7 (#3735), ORC6 (#4737) and Ddb1 (#5428) from Cell Signaling. Additional antibodies, chemiluminescence and densitometry are described in [18]. Immunoprecipitation antibodies were cyclin E1(C-19) from Santa Cruz Biotechnology and cyclin E2 (AbCam, [EP454Y]).

Densitometry of western blots was performed using ImageJ [47].

### 4.4. Flow Cytometry

Cells stained with 10 μg/mL propidium iodide (Sigma, Castle Hill, Australia) for 2–5 h and incubated with 0.5 mg/mL RNase A (Sigma) were analysed on a FACSCanto (BD Biosciences, North Ryde, Australia). Data were analysed using FlowJo (BD Biosciences). BrdU incorporation was detected with anti-BrdU-FITC (BD Biosciences), as previously described [17]. At least 20,000 events were analysed per replicate.

### 4.5. Microscopy

Live cell imaging on a Zeiss Axiovert 200 M inverted fluorescence microscope (10× objective; 0.3 N.A.), was performed at 37 °C with 5% CO_2_ and phase contrast images were captured every 5 min, as described in [48]. Multidimensional time-lapsed images were aligned using ImageJ software [47]. Mitotic duration was determined as the time from nuclear envelope breakdown to anaphase, with the protocol described in detail in [48].

### 4.6. siRNA Transfection

The siRNAs (Dharmacon, Lafayette, CO, USA) were transfected at 2–50 nM using Lipofectamine 2000 (Invitrogen, Carlsbad, CA, USA) for 24–72 h. Controls were: siControl Pool (D-001810-10), siCONTROL individual siRNAs (D-001810-1-4) and mock transfection. HeLa cells were transfected with 50 nM Ddb1 siRNAs (#6,7,9: Cat#J-012890-06-0020, J-012890-07-0020, J-012890-09-0020) and MCF-7 cells with 20 nM siRNAs to cyclin E1 (Cat#J-003213-10-13), cyclin E2 (Cat#L-003214-00) or cyclin A2 (Cat#L-003205-00).

### 4.7. TCGA (The Cancer Genome Atlas) Dataset and Statistical Analysis

Gene expression data were downloaded from the TCGA through cBioportal [49,50] in July 2017, Dec 2019 and July 2020.

Normalised individual 25-gene index of chromosomal instability (CIN25) gene expression data were extracted from TCGA datasets based on the median of all three available expression platforms. The CIN25 score was determined for each sample by the sum of 25 gene expression values of CIN genes [20].

ASCAT ploidy analyses and gene expression data were downloaded from COSMIC (v86, 14 Aug 18). Samples with estimated >40% tumour cellularity were taken forward for ploidy comparisons [51], where genome doubled tumours were defined as ≥2.7 ploidy [52], as confirmed by histogram analysis (Figure S1). Gene amplification status was defined based on GISTIC analysis, where +2 was equated to amplification and −2 to deep deletion. Samples amplified for both *CCNE1* and *CCNE2* (*n* = 5) were excluded from the analysis of ploidy and survival associated with gene amplification. For mRNA expression, the datasets were split into tertiles. High RNA expression was defined as the top tertile, and low expression defined as the bottom tertile.

Comparisons between the mRNA of *CCNE1*, *CCNE2*, *CCND1*, *CCND2*, *MKI67* and CIN25 scores in genome doubled versus non genome doubled breast cancers were performed with Welch’s unpaired *t*-tests, due to the unequal sample sizes in each group. Comparisons of the amplification status of *CCNE1*, *CCNE2*, *CCND1* and *CCND2* were performed using contingency analysis of the proportion of amplified samples in each group, using Fisher’s exact test.

The p53 functional cancers were defined as p53 wildtype cancers with high TP53 (z-score > 0), and high p21 (*CDKN1A)*. *CDKN1A* expression data were binned to identify the distribution of *CDKN1A* expression in the p53 wildtype and p53 mutated/deep deleted cancers (Figure S3). Using these distributions, we identified the overlap in *CDKN1A* expression between p53 mutated/deep deleted cancers and p53 wildtype breast cancers. Sixty-nine *TP53* wildtype breast cancers had a below median expression of *CDKN1A*, which corresponded to a low expression of *CDKN1A* in p53 mutated/deep deleted cancers, suggesting that these cancers could have compromised p53 function. These samples were then excluded to define a p53 wildtype/TP53 high/p21 (*CDKN1A)* high subset.

### 4.8. Survival Analyses

Survival analyses were performed using logrank Mantel–Cox tests on GraphPad Prism. Hazard ratios (logrank) were computed for each analysis, and reported along with the 95% confidence interval.

### 4.9. Statistics

Experiments were performed in triplicate, except where indicated. Data were analysed in GraphPad Prism, using one-way ANOVA or non-parametric Mann–Whitney tests, as appropriate. When one-way ANOVA was used, the differences between individual samples were compared using Tukey’s multiple comparisons test. For proportional analyses, a contingency analysis with a chi-squared test was performed. Error bars are standard error of the mean, except where indicated.

## 5. Conclusions

Overall, our data demonstrate that both cyclins E1 and E2 predict overall survival in non-genome doubled cancers, but the high expression of cyclin E1 or cyclin E2 is likely to provoke different events in the evolution of breast cancer. We provide evidence from in vitro models that cyclin E1 tends to induce mitotic slippage, whereas cyclin E2 induces genome rereplication (Figure 7). Finally, cyclin E2 amplification is highly prevalent (~16%) in breast cancers, indicating that pathways downstream of cyclin E2, including rereplication events, may affect the genesis of a significant proportion of breast cancers.

## Supplementary Materials

The following are available online at https://www.mdpi.com/2072-6694/12/8/2268/s1, Figure S1: Distribution of ploidy in breast cancers, Figure S2: Association of cyclin E1 with whole genome doubling in ovarian cancer. Figure S3: Expression of *CDKN1A* in *TP53* mutant/deleted and *TP53* wildtype/*TP53* high cancers. Figure S4: Uncropped western blot figures of Figure 3A and Figure 4D, Figure S5: Uncropped western blot figures of Figure 5C (Blot#1), Figure S6: Uncropped western blot figures of Figure 5C (Blot#2), Figure S7: Uncropped western blot figures of Figure 5C (Blot#3), Figure S8: Uncropped western blot figures of Figure 5E (cyclin E1 IPs), Figure S9: Uncropped western blot figures of Figure 5E (cyclin E2 IPs), Figure S10: Uncropped western blot figures of Figure 5G, Figure S11: Uncropped western blot figures of Figure 6F. Data sharing not applicable to this article as no datasets were generated during the current study. Vectors and cell line derivatives generated for this study are available upon request from the corresponding author.

## Abbreviations

CDK	cyclin-dependent kinase
CIN25	25-gene index of chromosomal instability
GD	genome doubled
GFP	green fluorescent protein
K-M	Kaplan–Meier
pMIG	pMSCV-IRES-GFP retroviral expression vector
preRC	pre-replication complex
MCM	minichromosome maintenance proteins
NGD	non-genome doubled
siRNA	small interfering RNA
TCGA	The Cancer Genome Atlas
2N	two sets of chromosomes
4N	four sets of chromosomes
8N	eight sets of chromosomes
