# Supplementary material for: Cyclin E2 Promotes Whole Genome Doubling in Breast Cancer
Source: Cancers (Basel). 2020 Aug 13;12(8):2268. doi: 10.3390/cancers12082268 (PMC7463708; doi:10.3390/cancers12082268)
Supplement: Supplementary file 1 [file cancers-12-02268-s001.pdf]

Supplementary Materials: Cyclin E2 Promotes Whole Genome Doubling in Breast Cancer

Christine Lee, Kristine Fernandez, Sarah Alexandrou, C. Marcelo Sergio, Niantao Deng, Samuel Rogers, Andrew Burgess, C. Elizabeth Caldon

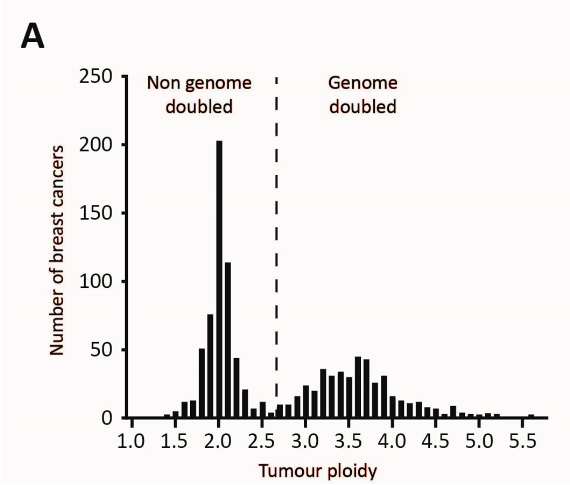

Figure S1. Distribution of ploidy in breast cancers. A. Binned histogram of TCGA breast cancers of >40% cellurity (n = 831) based on tumour ploidy. Dashed line indicates the cut-off between non genome doubled and genome doubled breast cancers.

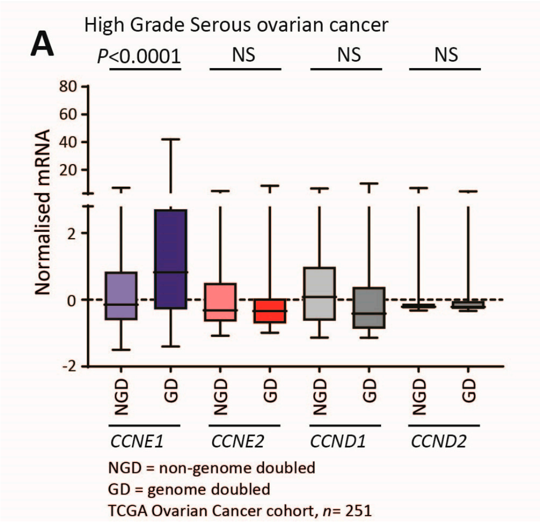

Figure S2. Association of cyclin E1 with whole genome doubling in ovarian cancer. A. Relative *CCNE1, CCNE2, CCND1, CCND2* expression was determined across the TCGA Ovarian Cancer dataset and compared between non-genome doubled (NGD) and genome doubled (GD) cancers. Data analysed by Welch's t-test.

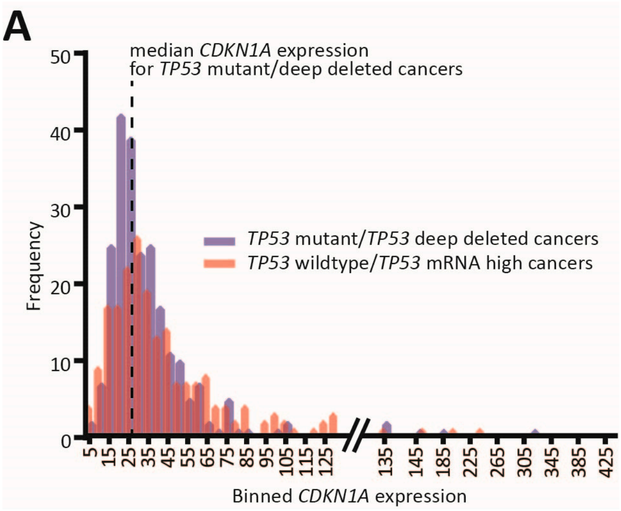

Figure S3: Expression of *CDKN1A* in *TP53* mutant/deleted and *TP53* wildtype/*TP53* high cancers A. p53 functional cancers were defined as p53 wildtype cancers with high *TP53* (Z-score>0), and high p21 (*CDKN1A*). *CDKN1A* expression data was binned to identify the distribution of *CDKN1A* expression in the p53 wildtype/*TP53* high (red) and p53 mutated/deep deleted cancers (blue). There was a lower median *CDKN1A* expression in p53 mutated/deep deleted cancers compared to p53 wildtype/*TP53* high breast cancers.

Figure 3A - lanes 1-3
Figure 4D - lanes 4-9

Lanes (right to left)

1 - EV UT - T-47D empty vector untreated
2 - E1 UT - T-47D cycE1 untreated
3 - E2 UT - T-47D cycE2 untreated
4 - EV Thy - T-47D empty vector thymidine
5 - E1 Thy - T-47D cycE1 thymidine
6 - E2 Thy - T-47D cycE2 thymidine
7 - EV 24h - T-47D empty vector 24h release
8 - E1 24h - T-47D cycE1 24h release
9 - E2 24h - T-47D cycE2 24h release
10 - EV 48h - T-47D empty vector 48h release
11 - E1 48h - T-47D cycE1 48h release
12 - E2 48h - T-47D cycE2 48h release
13 - BioRad Precision Plus Protein™ Dual Color Standard

MW = molecular weight

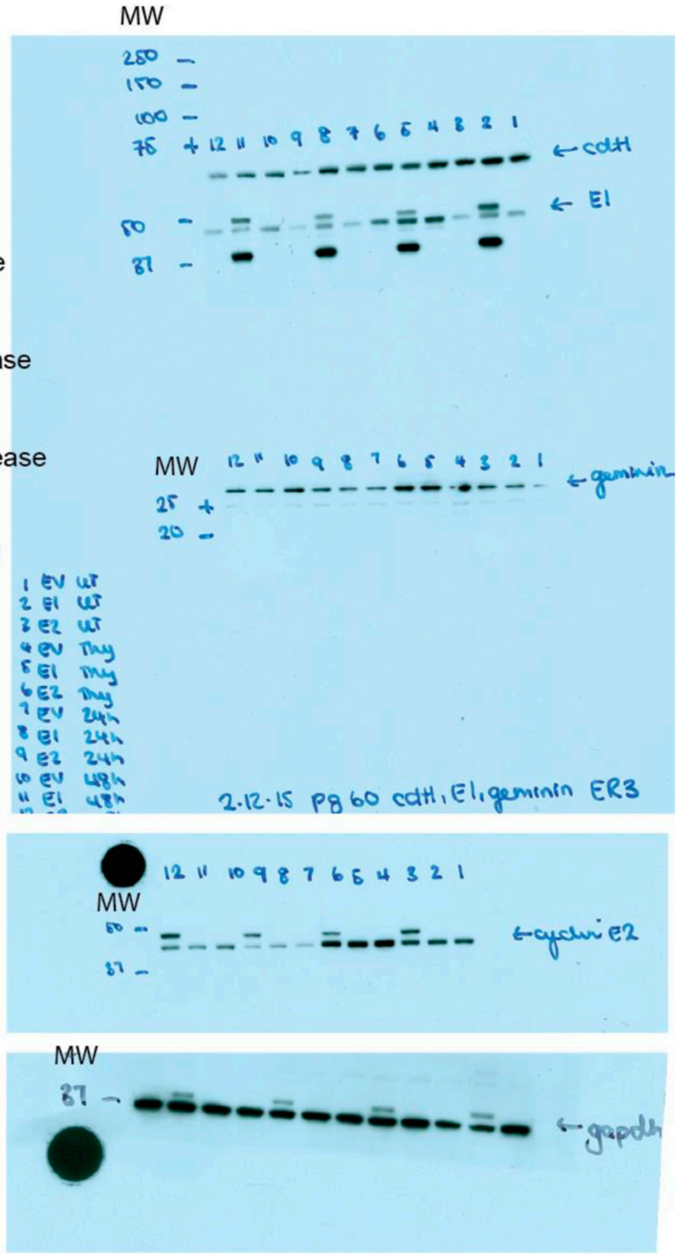

Densitometry (Image J)

LANE	1	2	3	4	5	6	7	8	9	10	11	12
Cdt1	100	127	93	101	69	82	72	87	22	55	48	42
cyclin E1	100	726	50	440	605	261	43	299	27	161	341	61
cyclin E2	100	104	203	183	166	199	8	19	63	58	33	162
GAPDH	100	50	59	101	97	91	89	75	80	104	121	119

Figure S4. Uncropped western blot figures of Figure 3A and Figure 4D.

Figure 5C (Blot#1): cytosolic, nuclear soluble and chromatin lysates of RO3306 treated HeLa cells

Lanes

1 - BioRad Precision Plus Protein™ Dual Color Standards
2 - untreated
3 - 24h thymidine block
4 - Oh RO3306
5 - 12h RO3306
6- 16h RO3306
7 - 20h RO3306
8 - 24h RO3306
9 - 24.5h RO3306
10 - Total cell lysate

MW = molecular weight
S1 = cytoplasmic lysate
S2 = nuclear soluble lysate
Chr = chromatin lysates

Densitometry (quantitated with ImageJ)

	LANE	2	3	4	5	6	7	8	9	10
cytoplasmic	MCM7	89	86	90	93	107	89	81	81	100
	Cdc6	7	51	12	41	24	3	1	1	100
	Cyclin E2	0	6	3	0	0	0	0	0	100
	CDK2	90	94	86	81	96	87	82	83	100
nuclear soluble	MCM7	66	57	65	86	105	104	109	104	100
	Cdc6	764	189	387	233	383	253	680	653	100
	Cyclin E2	0	0	0	0	0	0	70	70	50
	CDK2	60	113	128	108	120	117	120	134	100
chromatin	MCM7	94	115	119	99	59	45	100	118	100
	Cdc6	82	144	147	137	128	126	108	130	100
	Cyclin E2	93	44	36	4	0	1	78	172	100
	CDK2	120	106	109	109	118	124	110	128	100

normalised to total cell lysate (lane 10)

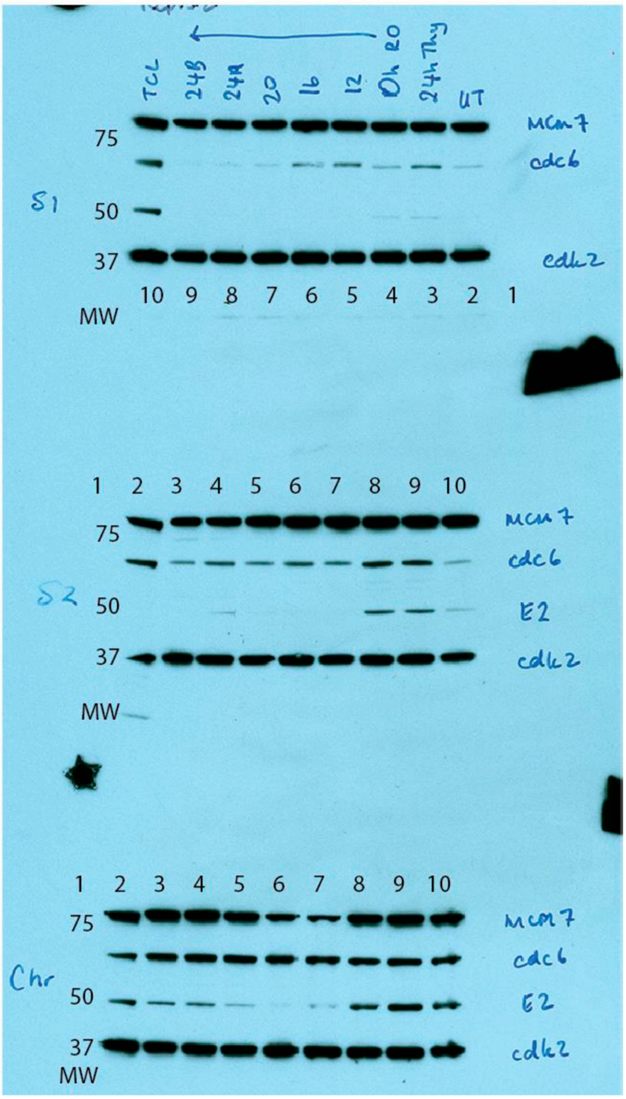

Figure S5. Uncropped western blot figures of Figure 5C (Blot#1).

Figure 5C (Blot#2): cytosolic, nuclear soluble and chromatin lysates of RO3306 treated HeLa cells

Lanes

1 - BioRad Precision Plus Protein™ Dual Color Standards
2 - untreated
3 - 24h thymidine block
4 - Oh RO3306
5 - 12h RO3306
6- 16h RO3306
7 - 20h RO3306
8 - 24h RO3306
9 - 24.5h RO3306
10 - Total cell lysate

MW = molecular weight
S1 = cytoplasmic lysate
S2 = nuclear soluble lysate
Chr = chromatin lysates

Densitometry (quantitated with ImageJ)

	LANE	2	3	4	5	6	7	8	9	10
cytoplasmic	MCM7	127	138	142	122	131	143	117	123	100
	Cdc6	9	79	49	73	33	21	10	6	100
	cyclin E1	44	119	87	12	10	17	35	42	100
	GAPDH	121	114	100	91	83	100	121	131	100
nuclear soluble	MCM7	1503	1896	2013	1976	1312	1178	1411	1009	50
	Cdc6	52	130	126	58	62	46	54	50	50
	cyclin E1	123	339	300	24	21	30	79	77	100
	GAPDH	9	31	20	18	10	8	16	11	100
chromatin	MCM7	166	180	116	30	51	127	140	144	100
	Cdc6	343	424	305	284	295	361	345	331	100
	cyclin E1	79	289	198	17	14	34	92	93	100
	GAPDH	8	20	10	14	9	7	14	13	100

normalised to total cell lysate (lane 10)

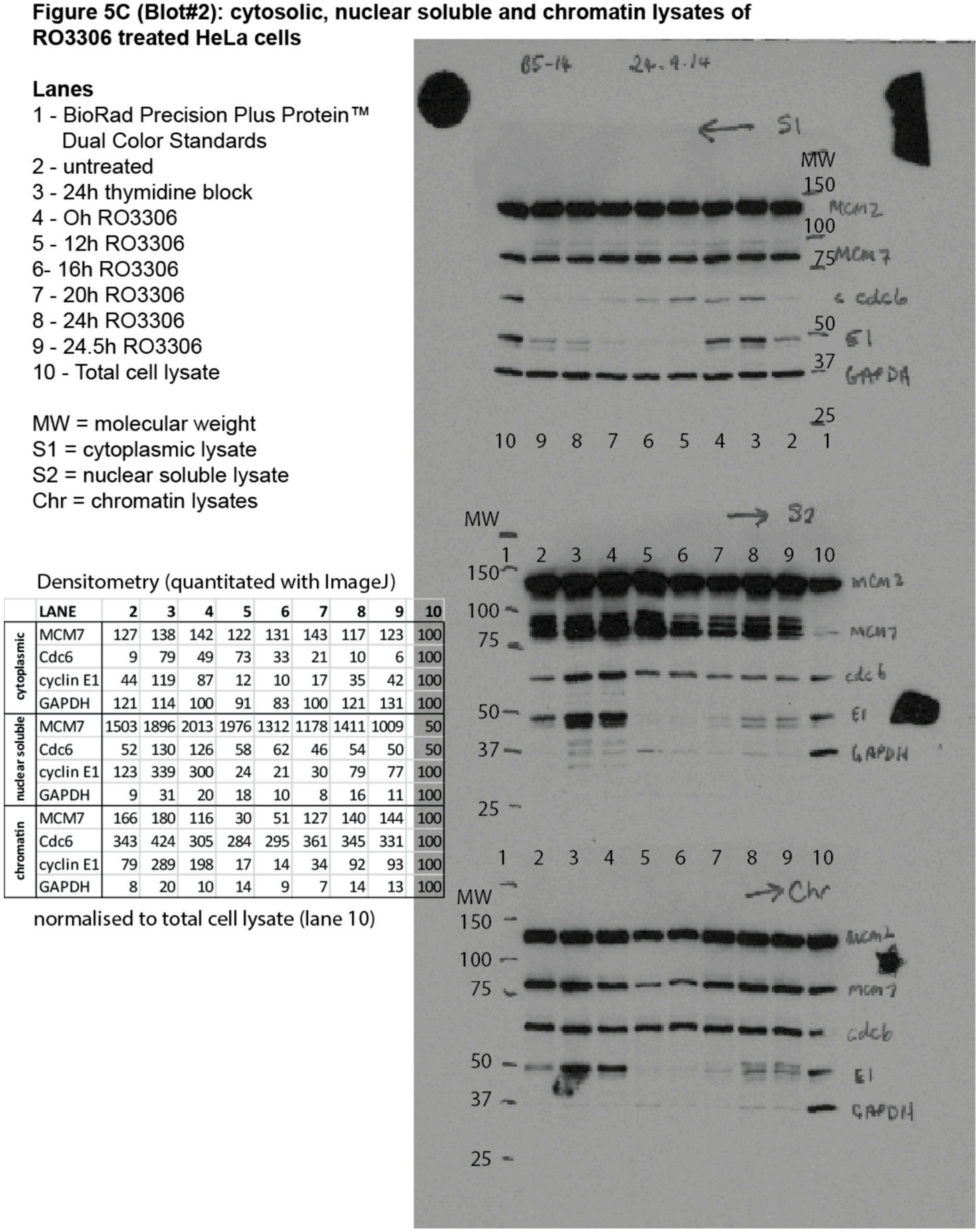

Figure S6. Uncropped western blot figures of Figure 5C (Blot#2).

Figure 5C (Blot#3): cytosolic, nuclear soluble and chromatin lysates of RO3306 treated HeLa cells

Lanes

1 - BioRad Precision Plus Protein Dual Color Standards
2 - untreated
3 - 24h thymidine block
4 - Oh RO3306
5 - 12h RO3306
6- 16h RO3306
7 - 20h RO3306
8 - 24h RO3306
9 - 24.5h RO3306
10 - Total cell lysate

MW = molecular weight
S1 = cytoplasmic lysate
S2 = nuclear soluble lysate
Chr = chromatin lysates

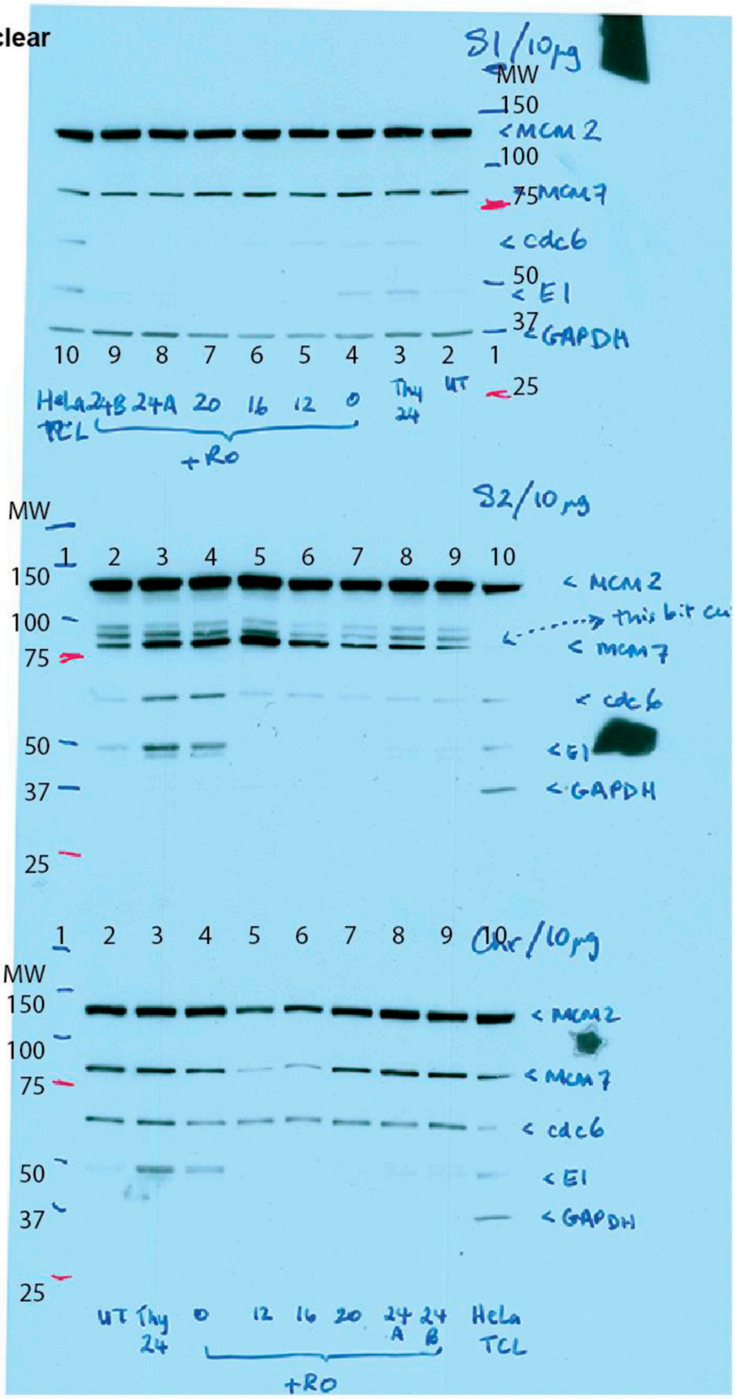

Densitometry (quantitated with ImageJ) normalised to total cell lysate (lane 10)

	LANE	2	3	4	5	6	7	8	9	10
cytoplasmic	MCM2	107	110	107	104	109	111	106	117	100
nuc sol	MCM2	188	207	199	224	178	163	185	167	100
chromatin	MCM2	95	102	95	27	52	86	109	92	100

Figure S7. Uncropped western blot figures of Figure 5C (Blot#3).

Figure 5E: cyclin E1 IPs - lanes 2 (IgG), lanes 6-7

Lanes (left to right)

1 - whole cell lysate of HeLa cells
2 - Chr 24h RO - cycE1 IP of chromatin extract treated wtih RO3306 for 24h
3 - Chr UT - cycE1 IP of chromatin extract untreated
4 - WCL 24h RO - cycE1 IP of whole cell lysate treated wtih RO3306 for 24h
5 - WCL UT - cycE1 IP of whole cell lysate untreated
6 - IgG IP control
7 - BioRad Precision Plus Protein™ Dual Color Standards

MW = molecular weight

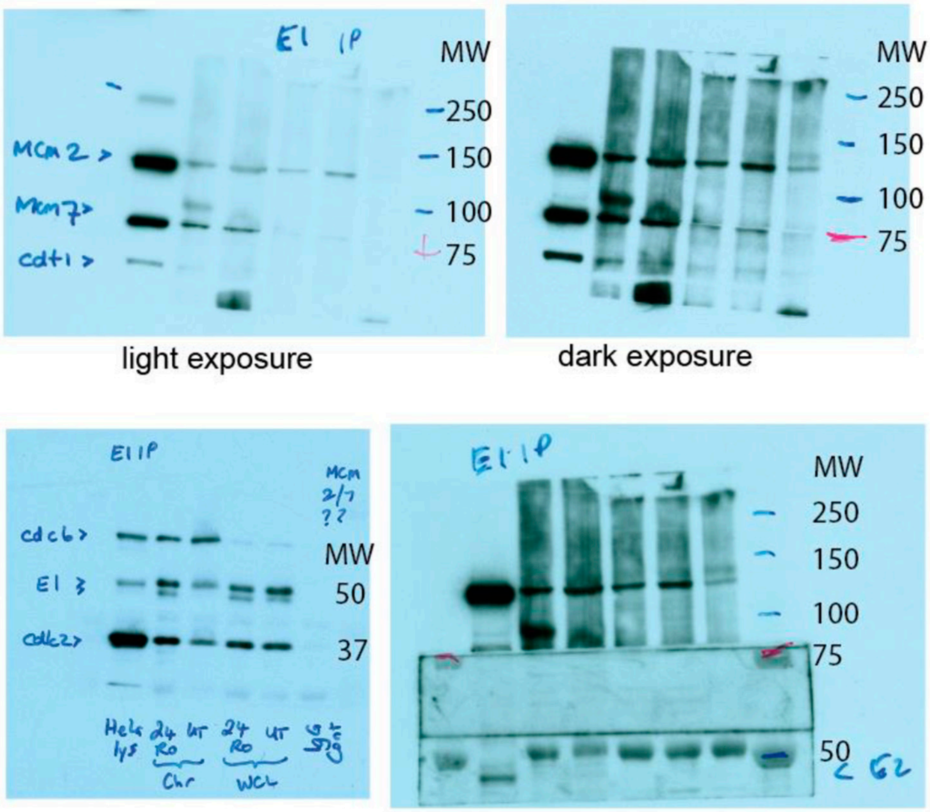

Densitometry (quantitated with ImageJ)

LANE	1	2	3	4	5
MCM2	100	7	6	1	3
MCM7	100	61	61	6	5
Cdt1	100	26	51	0	0
Cdc6	100	74	115	8	8
cyclin E1	100	444	121	331	486
Cdk2	100	32	9	24	20
cyclin E2	100	11	8	0	1

normalised to total cell lysate (lane 1)

Figure S8. Uncropped western blot figures of Figure 5E (cyclin E1 IPs).

Figure 5E: cyclin E2 IPs - lanes 2 (IgG), lanes 6-7

Lanes (right to left)

1 - BioRad Precision Plus Protein™ Dual Color Standards
2 - IgG IP control
3 - WCL UT - cycE2 IP of whole cell lysate untreated
4 - WCL 24h RO - cycE2 IP of whole cell lysate treated wtih RO3306 for 24h
5 - Chr UT - cycE2 IP of chromatin extract untreated
6 - Chr 24h RO - cycE2 IP of chromatin extract treated wtih RO3306 for 24h
7 - whole cell lysate of HeLa cells

MW = molecular weight

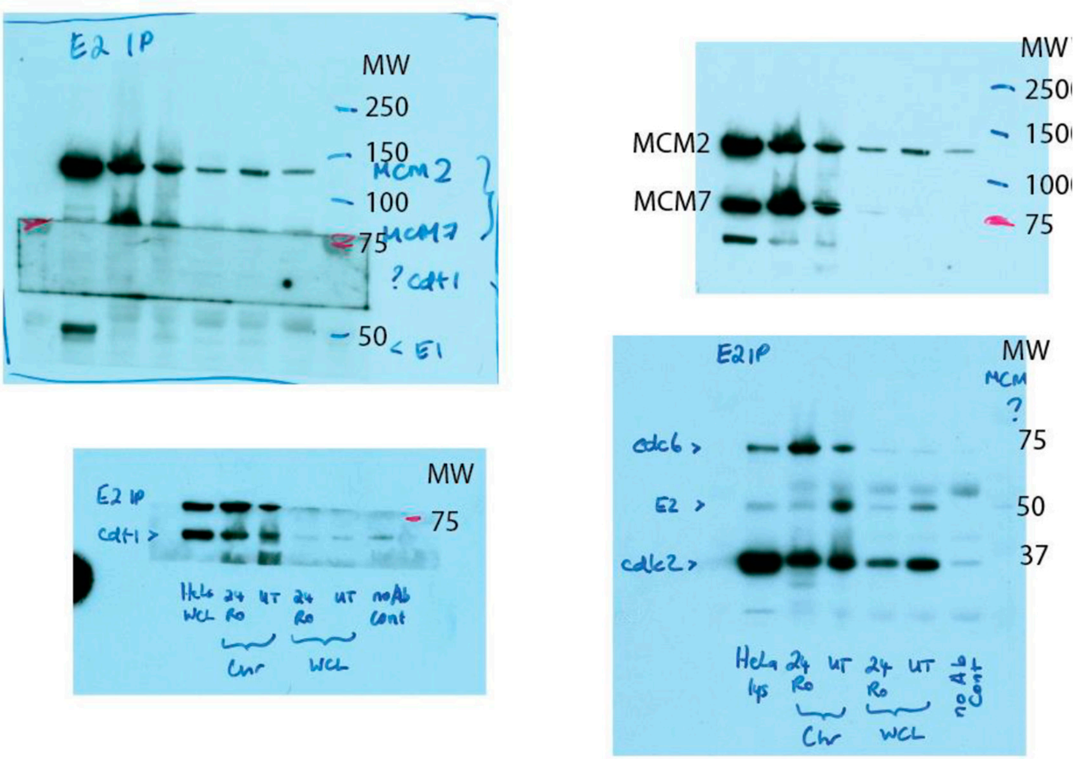

Densitometry (quantitated with ImageJ)

Lane	1	2	3	4	5	6
MCM2	100	71	25	4	8	0
MCM7	100	140	42	0	0	0
cyclin E1	100	0	0	0	0	0
Cdt1	100	65	41	0	0	2
Cdc6	100	1000	184	0	0	0
cyclin E2	100	75	2419	7	504	0
CDK2	100	54	44	13	33	0

normalised to total cell lysate (lane 1)

Figure S9. Uncropped western blot figures of Figure 5E (cyclin E2 IPs).

Lanes

1 - BioRad Precision Plus Protein™ Dual Color Standards
2 - UT = untreated
3 - NTP = non-targeting control
4 - E1 - cyclin E1 siRNA
5 - E2 - cyclin E2 siRNA
6 - Ddb1 - Ddb1 siRNA
7 - D + E1 - Ddb1 + cyclin E1 siRNA
8 - D + E2 - Ddb1 + cyclin E2 siRNA

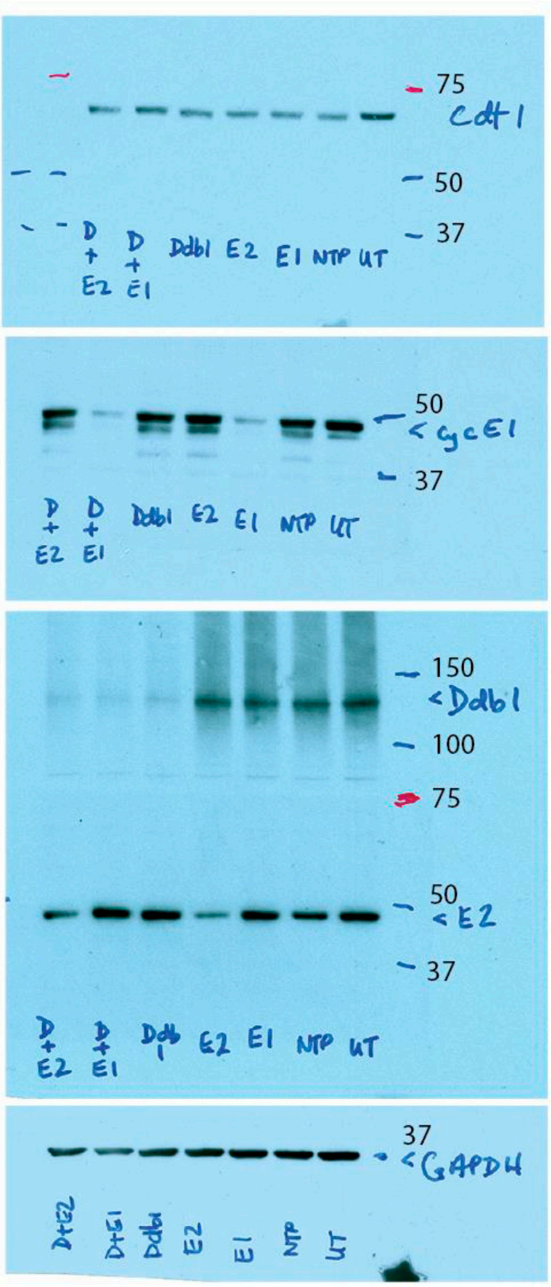

Densitometry (performed with ImageJ)
Data norrmalised to non-targeting siRNA treatment in lane 2

	1	2	3	4	5	6	7
**Cdt1**	415	100	113	124	146	221	143
**cyclin E1**	110	100	6	129	111	3	107
**Ddb1**	128	100	99	104	7	5	7
**cyclin E2**	159	100	148	23	174	169	58
**GAPDH**	121	100	100	88	75	39	61

Figure S10. Uncropped western blot figures of Figure 5G.

Figure 6F: lanes 4-10

Membranes were cut into strips get matching western blots without stripping the membrane. Films of the probed strips of membrane are shown.

Lanes (right to left)

1 - BioRad Precision Plus Protein™ Dual Color Standards
2 - Mock treatment
3 - Mock treatment
4 - UT - non-targeting control
5 - A - cyclin A2 siRNA
6 - E1 - cyclin E1 siRNA
7- E2 - cyclin E2 siRNA
8 - A + E1 - cyclin A2 siRNA + cyclin E1 siRNA
9 - A + E2 - cyclin A2 siRNA + cyclin E2 siRNA
10 - A + E1 + E2 - cyclin A2 siRNA + cyclin E1 siRNA + cyclin E2 siRNA

MW = molecular weight

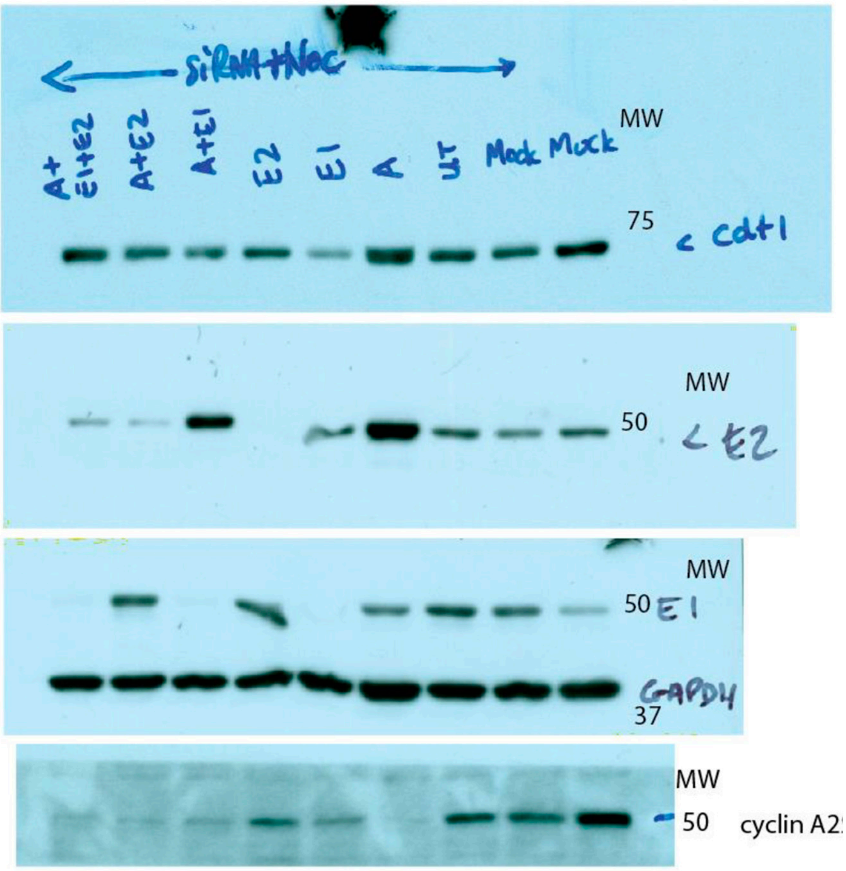

Densitometry (performed with ImageJ)
Data normalised to mock treatment in lane 2

LANE	2	3	4	5	6	7	8	9	10
Cdt1	226	100	135	207	10	97	50	83	155
cyclin E2	141	100	150	490	122	11	307	23	47
cyclin E1	16	100	155	74	0	163	0	124	1
GAPDH	102	100	105	135	96	87	83	85	83
cyclin A2	194	100	91	1	25	61	14	7	4

Figure S11. Uncropped western blot figures of Figure 6F.

© 2020 by the authors. Submitted for possible open access publication under the terms and conditions of the Creative Commons Attribution (CC BY) license (http://creativecommons.org/licenses/by/4.0/).
